# Reflecting on non-reflective action: An exploratory think-aloud study of self-report habit measures

**DOI:** 10.1111/bjhp.12060

**Published:** 2013-07-22

**Authors:** Benjamin Gardner, Vinca Tang

**Affiliations:** 1 Health Behaviour Research Centre, University College LondonUK; 2 Division of Psychology and Language Sciences, University College LondonUK

**Keywords:** Habit, self-report, think-aloud, measurement

## Abstract

**Objectives:**

Within health psychology, habit – the tendency to enact action automatically as a learned response to contextual cues – is most commonly quantified using the ‘Self-Report Habit Index’, which assesses behavioural automaticity, or measures combining self-reported behaviour frequency and context stability. Yet, the use of self-report to capture habit has proven controversial. This study used ‘think-aloud’ methods to investigate problems experienced when completing these two measures.

**Design:**

Cross-sectional survey with think-aloud study.

**Methods:**

Twenty student participants narrated their thoughts while completing habit measures applied to four health-related behaviours (active commuting, unhealthy snacking, and one context-free and one context-specific variant of alcohol consumption). Data were coded using thematic analysis procedures.

**Results:**

Problems were found in 10% of responses. Notable findings included participants lacking confidence in reporting automaticity, struggling to recall behaviour or cues, differing in interpretations of ‘commuting’, and misinterpreting items.

**Conclusions:**

While most responses were unproblematic, and further work is needed to investigate habit self-reports among larger and more diverse samples, findings nonetheless question the sensitivity of the measures, and the conceptualization of habit underpinning common applications of them. We offer suggestions to minimize these problems.

**Statement of contribution**

**What is already known on this subject?**

- Habit is most commonly measured within health psychology via the Self-Report Habit Index, or a combination of self-reported behaviour frequency and contextual stability.
- The suitability of self-report for capturing automatic processes has been questioned.

**What does this study add?**

- This is the first study of how people interpret and respond to self-report habit measures.
- Results show the potential for errors in recalling automaticity, cues, and behaviours.
- We discuss practical and theoretical challenges to assessing habit in health behaviours.

Habits have been defined as dispositions to automatically enact behaviours in specific contexts, acquired by learning context-behaviour associations (Ouellette & Wood, 1998). Habits form through repetition in stable contexts, which reinforces a mental context–behaviour association to the extent that encountering the context can automatically trigger the associated behaviour (Lally, van Jaarsveld, Potts, & Wardle, 2010; Wood & Neal, 2007). Whereas deliberative intentional action is cognitively effortful, habits proceed rapidly and efficiently, without awareness, control, or conscious intent (Bargh, 1994). Habitual tendencies are therefore thought to override competing intentions in determining behaviour in associated settings (Gardner, de Bruijn, & Lally, 2011; Triandis, 1977). Habitual performance reinforces the context–behaviour link, and so habits are self-sustaining over time (McGowan *et al*., in press[Bibr b24]). Habit thereby offers a mechanism for behaviour maintenance (Rothman, Sheeran, & Wood, 2009).

Development in habit theory and application requires robust habit measures. Automaticity is the ‘active ingredient’ of effects of habit on action (Gardner, Abraham, Lally, & de Bruijn, 2012). It has been argued that because automatic action occurs without conscious awareness (Bargh, 1994), self-reports of automaticity are unreliable (Eagly & Chaiken, 1993; Sniehotta & Presseau, 2012). Consequently, in self-report surveys, habit has traditionally been measured using past behaviour frequency (Triandis, 1977), but behaviour frequency alone cannot distinguish habitual from repeated deliberative action (Ajzen, 2002). Wood and colleagues developed a habit measure that multiplicatively combines (past or typical) behaviour frequency (‘how often do you do behaviour X?’) and context stability (‘when you do behaviour X, how often is cue Y present?’; Ouellette & Wood, 1998). Cues are researcher-selected and typically specified as physical location, time, other people, and mood; measuring all four cues and one frequency item generates four habit strength estimates (Ji & Wood, 2007). Applications of this ‘behaviour frequency × context stability’ (BFCS) measure have identified theorized effects of habit on action, predicting behaviour frequency and demonstrating an interaction with intention in guiding behaviour (Ji & Wood, 2007; Ouellette & Wood, 1998; Wood, Tam, & Witt, 2005). BFCS measures infer habit from the conduciveness of circumstances to habit development, but do not assess the automaticity with which behaviour is elicited.

The 12-item Self-Report Habit Index (SRHI; Verplanken & Orbell, 2003) was developed to capture reflections on three proposed facets of habit: automaticity ([‘Behaviour X is something…’] ‘…I do without thinking’), performance frequency (‘…I do frequently’), and relevance to self-identity (‘…that's typically ‘me’). The SRHI sidesteps concerns about self-reporting habit by focusing on common experiences of automaticity, such as efficiency (‘…I have no need to think about doing’), lack of awareness (‘…I start doing before I realize I'm doing it’), and uncontrollability (‘…I would find hard not to do’; Verplanken & Orbell, 2003). The SRHI has been shown to detect hypothesized habit-behaviour effects (Gardner *et al*., 2011), but its conceptual basis has been questioned (Gardner, Abraham *et al*., 2012; Sniehotta & Presseau, 2012). Identity is not an essential component of habit (Gardner, de Bruijn, & Lally, 2012), and frequency indicators in the SRHI inflate true effects of automaticity on action (Gardner, Abraham, *et al*., 2012). A subset of four automaticity SRHI items (the ‘Self-Report Behavioural Automaticity Index’; SRBAI) has been identified, which remains sensitive to expected effects (Gardner, Abraham, *et al*., 2012).

While BFCS and SRHI measures have been widely adopted (e.g., Gardner *et al*., 2011; Ji & Wood, 2007), their sensitivity to habitual action has been insufficiently investigated. Questions have been raised around the content validity of some SRHI items (Gardner, Abraham *et al*., 2012). It is unclear whether participants can reliably recall contextual covariates of behaviour in response to BFCS measures or have sufficient insight into automatic processes to respond to the SRHI. It has been suggested that the SRHI taps participants’ awareness *on reflection* that they were *not aware of initiating action at the time that it was performed* (Gardner, Abraham, *et al*., 2012), and so automaticity is inferred from its consequences (e.g., ‘I cannot recall lighting my cigarette, yet it is lit; therefore, I must have lit the cigarette automatically’; cf Sniehotta & Presseau, 2012). Yet, no empirical evidence is available to demonstrate thought processes among respondents to these measures.

‘Think-aloud’ procedures, whereby participants provide a spoken commentary of their thoughts during questionnaire completion, can reveal the reasoning, interpretations, and understandings that determine survey responses (French, Cooke, McLean, Williams, & Sutton, 2007). Recent applications of the ‘think-aloud’ method have highlighted its sensitivity to problems specific to the measures under study, such as misinterpretations and inconsistencies between respondents’ interpretations and their recorded responses, which may compromise reliability and validity (Darker & French, 2009; French *et al*., 2007; Kaklamanou, Armitage, & Jones, 2013). Findings can offer an empirical basis for refining measures, or highlight areas for consideration when applying them (Kaklamanou *et al*., 2013). ‘Think-aloud’ methods have not been used to examine habit self-reports, but may prove fruitful in documenting problems in participants’ comprehension of habit indices.

## The present study

This study used ‘think-aloud’ procedures to investigate potential problems in participants’ interpretations of SRHI and BFCS items. The reliability and validity of self-report measures depend on participants interpreting and responding to items as intended by the researcher. We tested these assumptions by exploring what people think about when they complete self-report habit measures, as applied to four behaviours, based on the analysis of qualitative ‘think-aloud’ commentaries.

## Method

### Participants

To achieve consistency with previous habit studies, which have mostly employed student samples (Gardner *et al*., 2011), participants were 20 native English-speaking, university students (13 women and seven men; four undergraduates and 16 taught postgraduates; age 18–35, *M* = 25 years, *SD* = 4), recruited via a poster on a UK university campus. No incentives were offered for participation. Psychology students were ineligible for the study to ensure questionnaire interpretation could not be biased by psychology training.

‘Think-aloud’ methodology does not impose sample size constraints, and we had no *a priori* expectations regarding the likely emergent themes, so were unable to anticipate the point of theoretical saturation. Following French *et al*. (2007), a sample of 20 participants was deemed likely to capture a broad range of problems.

### Questionnaire

Participants completed a 72-item questionnaire about four behaviours: ‘eating unhealthy snacks’, ‘commuting to university by public transport’, ‘drinking alcohol’, and ‘drinking a second alcoholic drink after my first alcoholic drink when in a pub or bar’. Multiple behaviours were addressed to identify problems relevant to a range of potential applications. Three behaviours were selected from published SRHI studies, which captured potentially unhealthy (‘drinking alcohol’, ‘eating unhealthy snacks’ [Adriaanse *et al*., 2010; Lucas, Alexander, Firestone, & Lebreton, 2008]) and healthy actions (‘commuting to university by public transport’, that is, active transport; Gardner, 2009). A fourth behaviour, constructed in response to calls for SRHI applications to incorporate contextual elements (Sniehotta & Presseau, 2012), referred to instances in which a specific behaviour (‘drinking a second alcoholic drink…’) followed a preceding behaviour (‘…after my first alcoholic drink…’) in a given setting (‘…in a pub or bar’). The latter items (hereafter, ‘drinking a second alcoholic drink’) were chosen to permit comparison with the context-free ‘drinking alcohol’ items. We focused on the juncture between finishing one drink and starting another because pilot work suggested that cued habit tendencies (to continue drinking) can feasibly override *a priori* intentions (to leave after one drink) at this point. We do not view the elaborate wording of the ‘drinking a second alcoholic drink’ items as problematic, given previous SRHI applications specified at a similar level of complexity (Eccles *et al*., 2011; Rhodes, de Bruijn, & Matheson, 2010).

The 12 SRHI items followed a stem (‘[e.g., Eating unhealthy snacks] is something…’ ‘…I do automatically’) and were measured on a 1–7 scale on which three points were labelled (1 = *strongly disagree,* 4 = *neither agree nor disagree, and* 7 = *strongly agree*). Four SRHI items (numbered 2, 3, 5, and 8 in Table 1) comprise the SRBAI. Reliability was generally satisfactory for the SRHI (α range: .76–.90). SRBAI reliability was questionable for commuting (α = .65), but acceptable for the remaining behaviours (α range: .73–.76; Kline, 1999).

**Table 1:** Distribution of problem segments across problem types, items, and behaviours (snacking [S], commuting [C], alcohol [A], drinking a second alcoholic drink [SAD])

	Problem types						
	Interpretation				Sources of bias		
Item	Comprehension difficulty	Uncertain of appropriate response	Deviation from intended meaning	Contextual qualification	Self-presentation	Recall error	Totals
*SRHI:* (*‘Behaviour X is something…’*)							
1. I do frequently	0	1 (*A =* 1)	0	3 (*S =* 1*, SAD* = 2)	0	0	4 (*S =* 1*, A =* 1*, SAD* = 2)
2. I do automatically*	3 (*A =* 1*, SAD* = 2)	0	3 (*S =* 2*, C =* 1)	4 (*S =* 1*, A =* 2*, SAD* = 1)	1 (*A =* 1)	0	11 (*S =* 3*, C =* 1*, A =* 4*, SAD* = 3)
3. I do without having to consciously remember*	4 (*S =* 1*, A =* 2*, SAD* = 1)	2 (*S =* 1*, SAD* = 1)	7 (*S =* 2*, C =* 3*, SAD* = 2)	2 (*C =* 1*, A =* 1)	0	0	15 (*S =* 4*, C =* 4*, A =* 3*, SAD* = 4)
4. That makes me feel weird if I do not do it	0	0	6 (*C =* 1*, A =* 3*, SAD* = 2)	2 (*A =* 2)	1 (*A =* 1)	0	9 (*C =* 1*, A =* 6*, SAD* = 2)
5. I do without thinking*	0	0	0	1 (*SAD* = 1)	0	0	1 (*SAD* = 1)
6. That would require effort not to do	2 (*C =* 1*, SAD* = 1)	1 (*C =* 1)	2 (*C =* 1*, A =* 1)	3 (*S =* 1*, SAD* = 2)	1 (*A =* 1)	0	9 (*S =* 1*, C =* 3*, A =* 2*, SAD* = 3)
7. That belongs to my [daily/weekly/usual] routine	2 (*SAD* = 2)	0	3 (*A =* 1*, SAD* = 2)	2 (*S =* 1*, SAD* = 1)	0	0	7 (*S =* 1*, A =* 1*, SAD* = 5)
8. I start doing before I realize I'm doing it*	1 (*C =* 1)	1 (*SAD* = 1)	3 (*C =* 3)	0	0	0	5 (*C =* 4*, SAD* = 1)
9. I would find hard not to do	0	0	1 (*C =* 1)	2 (*S =* 1*, SAD* = 1)	0	0	3 (*S =* 1*, C =* 1*, SAD* = 1)
10. I have no need to think about doing	9 (*S =* 1*, A =* 6*, SAD* = 2)	1 (*S =* 1)	4 (*S =* 1*, C =* 1*, A =* 1*, SAD* = 1)	2 (*S =* 1*, SAD* = 1)	0	0	16 (*S =* 4*, C =* 1*, A =* 7*, SAD* = 4)
11. That's typically ‘me’	1 (*C =* 1)	0	4 (*S =* 2*, C =* 1*, A =* 1)	0	1 (*A =* 1)	0	6 (*S =* 2*, C =* 2*, A =* 2)
12. I have been doing for a long time	1 (*A =* 1)	3 (*A =* 1*, SAD* = 2)	4 (*S =* 1*, C =* 1*, SAD* = 2)	1 (*S =* 1)	0	0	9 (*S =* 2*, C =* 1*, A =* 2*, SAD* = 4)
*BFCS*							
Typical behaviour frequency	0	0	0	0	0	3 (*S =* 2*, C =* 1)	3 (*S =* 2*, C =* 1)
Actual past behaviour frequency	0	0	0	0	0	8 (*S =* 4*, C =* 2*, A =* 2)	8 (*S =* 4*, C =* 2*, A =* 2)
Mood cue	2 (*A =* 1*, SAD* = 1)	3 (*S =* 1*, C =* 2)	0	3 (*C =* 1*, A =* 1*, SAD* = 1)	0	0	8 (*S =* 1*, C =* 3*, A =* 2*, SAD* = 2)
People cue	1 (*SAD* = 1)	0	10 (*C =* 9*, A =* 1)	0	0	0	11 (*C =* 9*, A =* 1*, SAD* = 1)
Location cue	3 (*SAD* = 3)	1 (*A =* 1)	16 (*S =* 2*, C =* 10*, A =* 1*, SAD* = 3)	0	0	1 (*S =* 1)	21 (*S =* 3*, C =* 10*, A =* 2*, SAD* = 6)
Time cue	0	0	1 (*A =* 1)	2 (*A =* 2)	0	1 (*S =* 1)	4 (*S =* 1*, A =* 3)
*Totals*	29 (*S =* 2*, C =* 3*, A =* 11*, SAD* = 13)	13 (*S =* 3*, C =* 3*, A =* 3*, SAD* = 4)	64 (*S =* 10*, C =* 33*, A =* 10*, SAD* = 12)	27 (*S =* 7*, C =* 2*, A =* 8*, SAD* = 10)	4 (*A =* 4)	13 (*S =* 8*, C =* 3*, A =* 2)	150

*Note*. SRHI = Self-Report Habit Index; SRBAI = Self-Report Behavioural Automaticity Index; BFCS = Behaviour Frequency × Context Stability measure.

SRHI items are numbered for reference purposes; see main text. Numbers within the main cells represent total problems coded into each problem type for each item. Numbers in parentheses indicate behaviours for which problems were identified, where S = items relating to ‘eating unhealthy snacks’; C = ‘commuting by public transport’; A = ‘drinking alcohol’; SAD = ‘drinking a second alcoholic drink after my first when in a pub or bar’.

* SRHI items included within the SRBAI.

Behaviour frequency × context stability measures incorporated typical and actual past behavioural frequency and contextual cue items. *Typical behaviour frequency* was measured with a single-item (for drinking alcohol, snacking, and commuting: ‘In a typical week, how often do you [e.g., drink alcohol]?’ [1 *= rarely or never,* 2 *= about once a week,* 3 = *once every* 2*–*3 *days, and* 4 = *most or all days*]; for the context-specific behaviour: ‘When in a pub or bar, how often do you typically drink a second alcoholic drink after your first alcoholic drink?’ [1 = *rarely or never,* 2 = *sometimes,* 3 = *most of the time, and* 4 = *always*]). *Actual past behaviour* was measured via a single-item (‘on how many of the past 7 days did you [e.g., commute to university by public transport]?’; 0–7 days). Four *contextual cues* were assessed (mood, time of day, location, presence of others), each via a single statement (e.g., ‘when I drink alcohol, I am [1 = *rarely or never,* 2 = *sometimes,* 3 = *most of the time,* 4 = *always*] in the same mood’). A fifth option (*‘not applicable – I never do this’*), included to allow for the possibility that the behaviour was not relevant, was not selected by any participant for any behaviour.

### Procedure

Prior to questionnaire completion, participants read instructions adapted from the study by French *et al*. (2007): ‘We want to examine how you interpret questions commonly used in health-related research studies. We are going to ask you to fill in a questionnaire and “think aloud” as you fill it in. What we mean by “think aloud” is that we want you to say everything you are thinking, from the time you first see each question until you reach a decision on how to answer the question […] as if you were alone in the room speaking to yourself’ (abbreviated to avoid repetition; full instructions available on request). Participants first practised ‘thinking aloud’ in response to Theory of Planned Behaviour items (Ajzen, 2006) pertaining to ‘exercising regularly’.

During questionnaire completion, the researcher sat out of view and spoke only to remind the participant to continue talking if they fell silent for 10 s. ‘Think-aloud’ narratives were digitally recorded and transcribed verbatim.

### Analyses

Descriptive statistics for the habit indices were computed for sample description purposes. BFCS scores were computed by multiplying typical behaviour frequency and each contextual cue score, generating scores between 1 (no habit) and 16 (strong habit). SRHI and SRBAI values represented mean scale scores (1 = *no habit*, 7 = *strong habit*).

‘Think-aloud’ data were coded by both authors, using thematic analysis techniques. Each verbal item response was treated as a unitary ‘segment’, so generating 1,440 potentially codeable segments (72 items × 20 participants). The purpose of the analysis was to identify ‘problem segments’ – that is, responses suggesting interpretation difficulties, biased responding, or dissatisfaction with items (Kaklamanou *et al*., 2013) – and categories of ‘problem types’ into which these could be sorted. A coding frame of problem types, adapted from previous studies (Darker & French, 2009; French *et al*., 2007; Kaklamanou *et al*., 2013), was applied and iteratively refined to capture the content of all problem segments. Agreement between coders was 95.1%. Disagreements were resolved through discussion. Neither coder identified segments that fitted multiple problem types, and so each segment was allocated to only one problem type. The distribution of problem segments was tabulated to indicate the concentration of problem types across items (see Table 1) and the four behaviours.

Proper interpretations of SRHI items, which are designed to measure ‘repetition, automaticity (lack of control and awareness, efficiency), and … identity’ (Verplanken & Orbell, 2003, p. 1313), were based on extant literature where possible. Verplanken and Orbell (2003) specified that items 1, 7, and 12 pertain to frequency, and Honkanen, Olsen, and Verplanken (2005) identified items 3, 4, and 10 as respective indicators of lack of awareness, lack of control, and mental efficiency. Intended meanings of the remaining six items were inferred, prior to data collection, by the first author, and verified after analysis via comparison with those of two independent coders, both first authors of SRHI applications. Strong inter-rater consistency was found (coder 1: 100% agreement, ĸ = 1.0; coder 2: 83% agreement, ĸ = .78), with complete agreement for items 2 (general automaticity), 5 (lack of awareness), 6 (lack of control), 9 (lack of control), and 11 (identity), although item 8 was interpreted by the first author and one coder as ‘lack of control’, but by the other coder as ‘mental efficiency’.

## Results

### Descriptives

Commuting by public transport (*M* = 4.70 days per week) and snacking (*M* = 3.45 days) were the more frequently performed behaviours (Table 2). On average, participants drank alcohol on 1.80 days per week, and a second alcoholic drink in a pub or bar on 1.15 days. Mean scores suggested weak or no habit for snacking and drinking alcohol, and moderate habits for drinking a second alcoholic drink. For commuting, SRHI, SRBAI, and BFCS people and mood scores were below the scale midpoint, but above-midpoint scores suggested stronger location- and time-cued habits. Of BFCS measures, highest scores were observed for the mood-based measure for snacking and time-based measures for alcohol and drinking a second alcoholic drink.

**Table 2:** Descriptive statistics for habit indices, all behaviours

	Eating unhealthy snacks		Commuting to university by public transport		Drinking alcohol		Drinking a second alcoholic drink…	
Measure	Minimum–maximum	Mean (*SD*)	Minimum–maximum	Mean (*SD*)	Minimum–maximum	Mean (*SD*)	Minimum–maximum	Mean (*SD*)
*SRHI-based*								
SRHI	1–5.42	3.36 (1.35)	1.33–6.50	3.89 (1.53)	1–4.42	2.72 (.97)	1–5.08	2.97 (1.20)
SRBAI	1–5.25	2.83 (1.42)	1.00–6.25	3.24 (1.57)	1–4.25	2.18 (1.09)	1–4.75	2.34 (1.22)
*Behaviour frequency-based*								
Typical behaviour frequency	1–4	2.85 (.99)	1–4	3.05 (1.28)	1–4	1.90 (.85)	1–4	3.10 (1.02)
Actual past behaviour frequency	0–7	3.45 (2.11)	0–7	4.70 (1.87)	0–7	1.80 (1.91)	0–6	1.15 (1.60)
BFCS: Location*	1–12	5.35 (3.18)	1–16	9.60 (5.62)	1–12	4.15 (2.89)	1–16	7.90 (4.59)
BFCS: Time*	1–12	4.80 (2.69)	1–16	8.45 (4.44)	1–12	4.75 (2.83)	1–16	8.15 (4.34)
BFCS: People*	1–16	4.90 (3.65)	1–16	5.25 (4.51)	1–12	4.70 (2.94)	1–12	7.80 (3.33)
BFCS: Mood*	1–16	6.30 (3.73)	1–16	6.00 (4.10)	1–9	3.95 (2.42)	1–16	7.60 (4.26)

*Note*. SRHI = Self-Report Habit Index; SRBAI = Self-Report Behavioural Automaticity Index; BFCS = behaviour frequency × context stability.

* BFCS scores calculated by multiplying typical behaviour frequency and cue scores. Descriptives for cues are not reported.

Eighteen participants (90%) generated at least one problem segment, with 150 problems identified in total (10.4% of all segments; mean 7.89 problems per participant, *SD* = 4.99). Of these, 95 problems (63.3% of problem segments; generated by 17 participants) related to the SRHI, 32 (20.6%; 14 participants) of which applied to the SRBAI. SRHI items focusing on drinking a second alcoholic drink (30 problem segments) yielded more problems than for drinking alcohol (28), commuting (21), or snacking (19). Across the four behaviours, the SRHI item ‘[Behaviour X is something…] I have no need to think about doing’ was most problematic (16). All SRHI items generated at least one problem, yielding a mean of 1.98 problems per item per behaviour (*SD* = 4.56; SRBAI *M* = 2.00, *SD* = 6.22).

Fifty-five BFCS problem segments (36.7%; 16 participants) were coded, of which 52 related to cue items and 3 to behaviour. Twenty-five problems were linked to commuting (24 to cues, one to behaviour), 11 to snacking (nine cues, two behaviour), 10 to alcohol (cues), and 9 to (cues to) drinking a second alcoholic drink. Location cue items were most problematic (21 segments in total). At least three problems were coded for each BFCS item (mean 1.53 problems per item per behaviour, *SD* = 6.49).

### Problem types

Six problem types were identified. Four related to interpretation problems (comprehension difficulty, uncertainty of appropriateness of response, deviation from intended meaning, and contextual qualification), and two to sources of bias or error (self-presentation and recall error; see Table 1).

#### Interpretation problems

##### Comprehension difficulty

This category accounted for 29 problems (19.3% of all problems), generated by 12 participants, where the participant was unable to confidently comprehend intended item meanings and could not resolve this by rereading items. The majority (22) of comprehension problems related to the SRHI, particularly those featuring a negative clause (e.g., ‘… I have no need to think about doing’ [9 problems]; … ‘I do without having to consciously remember’ [4]). One participant found it difficult to decipher whether ‘drinking a second alcoholic drink’ SRHI items related to generic or context-specific performances:

‘Drinking a second alcoholic drink … is something that belongs to my usual routine.’ I'm not sure. I think it's something that I do frequently, but drinking alcohol is something that I do infrequently, almost never. I consistently drink a second drink *if I'm in a bar*, even if it's like three times a year, so… my drinking routine *in a bar*, yes, I guess. This question is kind of hard to answer. (Participant 14 [P14]; emphasis added)

In most instances, comprehension problems prompted scale midpoint responses (e.g., ‘I guess this one would be ‘neither agree nor disagree’, since I don't really understand what it is asking’, P7), although one participant deemed disagreement more appropriately (‘Huh? I'm not really sure about that question. Strongly disagree’; P15).

##### Uncertainty of appropriateness of response

Responses in this category (13 problems [8.7%]; nine participants) were those in which the participant understood the gist of an item, but doubted the suitability of their responses. In two instances, uncertainty arose from subjective wording:

‘Drinking alcohol is something I do frequently’. That's relative. For some people, once a week is frequent, for some people once a week is infrequent. (P3)

‘Drinking alcohol is something I have been doing for a long time’. I've been drinking now for five or six years. I don't know if that's ‘a long time’. (P9)

Three participants lacked confidence in reflecting on automaticity (e.g., ‘“Drinking a second alcoholic drink … is something I start doing before I realize I'm doing it.” I don't know if I could answer that’, P17; ‘I don't know if I think about it’, P14), and three people felt unable to reliably identify cues (e.g., ‘I'm not sure what mood I'm in when I go to university’, P10).

##### Deviation from intended meaning

This category was most populated (64 problems [42.7%]; 16 participants) and captured instances in which interpretations differed to those intended by researchers. Notable deviations for SRHI items included the belief that an item intended to tap non-awareness in initiating habitual action (‘[Behaviour X is something] … I do without having to consciously remember’) assessed whether behaviour could be reliably recollected (e.g., ‘if I had a second drink I'd probably remember’, P16; ‘I don't usually keep track of when I eat unhealthy snacks’, P4). An item designed to capture anomalous and unsettling experiences of non-performance of a habitual response (‘…that makes me feel weird if I do not do it’) was interpreted by four participants to assess awkwardness arising from social exclusion where applied to alcohol consumption:

I guess if you're at a party or a pub or when everyone else is drinking, it feels a little weird to be the one person not to do it. (P9)

For two participants, an item intended to capture mental efficiency (‘… I have no need to think about doing’), applied to snacking, evoked value judgements around whether people should be more mindful of their diet (‘I disagree with that, one should think about the snacks they're consuming’, P16). One participant interpreted this item to capture personal relevance of the behaviour (‘I don't need to think about [commuting to university using public transport] because I use the bike’, P11).

Other notable excerpts included one participant reading ‘automaticity’ to specify only innate reflexive actions (‘What do you mean by ‘[I do] automatically,’ do I do it unconsciously like breathing, like I'm conscious or unconscious of my heart beating?’ P16). Another interpreted an item about the compulsive nature of habitual action (‘… I would find hard not to do’), where applied to commuting, to capture practical difficulties associated with alternative transport options (‘I'd have to agree with that, in the sense that I'd have to find an alternative method of transportation, but it's possible – I could get a bicycle or walk’, P14).

Behaviour frequency × context stability cue items, which are designed to capture the contexts in which a habitual action commences, evoked most misinterpretations for commuting. Participants variously interpreted ‘commuting’ to begin when leaving home, when waiting for a bus or train, or when aboard the vehicle:

[I am in the same physical location] practically always, if it's from home. (P19)

Yeah, I always get the bus at the same place. (P7)

The whole point of public transport is to get you from one physical location to another, so I'd say never, I'm always moving. (P3)

One participant deviated from intended meanings by answering some items in relation to other people rather than herself:

‘Drinking alcohol is something that would require effort not to do’. That is a tough question, ‘cause I'm answering it objectively. For some people, where alcohol becomes a kind of … social tool, custom or norm, that'd be hard not to do. (P16)

##### Contextual qualification

This category encapsulated 27 instances (18.0%; 10 participants) in which participants provided responses based on contextual information not present within the item, or otherwise voiced concern over a lack of contextualization. The former resulted in differences between participants in interpretations of the same item, for example ‘Drinking alcohol is something I do automatically’:

Well, being in a social situation, when I'm going to be with friends, it's kind of automatic, in a sense. (P17)

I guess I'd slightly agree. If I go to a pub or club or something like that, I do it automatically then. It's not something like, when I get home, I'd have a drink right away, but it's automatic when I'm in a pub. (P9)

Contextual qualification was observed across all behaviours, even for items relating to drinking a second alcoholic drink, which explicitly specified location and preceding action cues:

‘Drinking a second alcoholic drink … is something I would find hard not to do’. A bit, I guess, yes, when everyone else is drinking. (P6)

Some participants gave neutral responses to items deemed insufficiently contextualized, despite indicating that they (dis)agreed with statements in certain settings. For example, a participant visiting the UK stated:

‘Eating unhealthy snacks is something I do frequently’. I'd say neither agree nor disagree. Now that I'm living on my own, I don't do that frequently, but when I'm … back home, I do it much more frequently ‘cause I know there'll be lots of chips and cookies around. (P9)

#### Sources of bias and error

##### Self-presentation

All four segments (2.7%; four participants) coded into this category arose from SRHI items relating to ‘drinking alcohol’, reflecting concerns that agreement with statements about habitual drinking would portray participants in a negative light (e.g., ‘would I sound like an alcoholic if I said “agree”?’; P3).

##### Recall error

This category (13 responses [8.9%]; 10 participants) captured difficulties participants experienced in accurately recalling their behaviour (11 responses) or cues (2), for example:

‘On how many of the past 7 days did you eat unhealthy snacks?’ I don't know, I can't count, so let's say three or four days, I don't know. (P17)

‘When I eat unhealthy snacks, I am in the same physical location’. I don't remember where I eat unhealthy snacks. (P13)

## Discussion

This study used ‘think-aloud’ methods to investigate potential difficulties in participants’ responses to two self-report measures of habitual action – the SRHI (Verplanken & Orbell, 2003) and measures combining behavioural frequency and context stability (BFCS; Ouellette & Wood, 1998) – as applied to four behaviours. Problems were identified in 10% of responses, and 90% of participants generated at least one problematic response. A minority of responses were problematic, and sample limitations call for investigation of habit self-reports among larger and more diverse populations. Nonetheless, findings point to the potential for comprehension and recall problems to compromise habit strength estimates. Some identified problems are generic to social cognition questionnaire completion, such as misinterpreting items to relate to others and not oneself (e.g., Kaklamanou *et al*., 2013), and may perhaps be addressed through clearer instructions to participants. Others reveal problems inherent to habit indices and may necessitate methodological and conceptual refinements.

The validity of self-report habit measures has been assumed based on convergence with other habit indices and on prediction of self-reported behaviour (Gardner, Abraham, *et al*., 2012; Ouellette & Wood, 1998; Verplanken & Orbell, 2003). However, these criteria overlook how respondents perceive and respond to items (Kaklamanou *et al*., 2013). We observed a number of interpretation problems specific to the studied measures. SRHI items designed to assess the efficiency and uncontrollability of habitual action were misinterpreted by some to capture ease of recalling behaviour, or whether people ought to think more about their actions. Some of these problematic items feature in the SRBAI subscale of the SRHI (Gardner, Abraham, *et al*., 2012), and so adopting the SRBAI instead of the SRHI may reduce but not remove interpretation errors. Some difficulties arose only in certain behaviour domains. For example, self-presentation concerns around classifying one's behaviour as automatic were raised only in relation to drinking, perhaps because alcohol consumption questions are prone to impression management biases (Davis, Thake, & Vilhena, 2010). Interpretations of habit items may depend on the behavioural domain within which they are framed. Studies of unhealthy or socially undesirable behaviours might benefit from a pilot phase, to identify and minimize self-presentation concerns.

Concerns have been raised that people cannot reliably reflect on habits, because habits proceed outside of awareness (Eagly & Chaiken, 1993). Some participants felt unable to accurately judge whether behaviour was automatic or deliberative and struggled to recall behaviour or environmental cues. Indeed, people lack insight into the psychological and environmental determinants of their behaviour (Nisbett & Wilson, 1977). Our findings question the utility of self-report habit measures. It may be unrealistic to expect participants to be attentive to actions undertaken with minimal deliberative input, or their contextual covariates. However, insight problems were observed only for some participants, behaviours, and cues. It may be that some habitual actions proceed less mindfully than others (Wood, Quinn, & Kashy, 2002), some contextual cues are less salient in memory, or individual differences exist in the ability to recall cognitions and environments. For example, people may be less attentive to time-based than to event-based cues (McDaniel & Einstein, 2000). Habits are a form of cue-dependent automaticity (Orbell & Verplanken, 2010), and so, as one participant noted, the frequency with which a contextual cue (a bar) automatically elicits a habitual response (drinking alcohol) will depend upon the frequency with which the cue is encountered (Gardner, 2012). Infrequent habitual actions may perhaps be harder to reflect upon. For these reasons, it is unclear whether the insight problems we observed generalize across actions and settings. More work is needed to assess the convergence of self-reported habit with objective automaticity indicators, such as those based on response times (Verplanken, Myrbakk, & Rudi, 2005).

Our findings contribute to ongoing debate around operationalizing habit in survey research. Questions have been raised around whether and how to combine the automaticity of the SRHI with the cue dependence of the BFCS. Some commentators have called for SRHI applications to specify both an action and the setting in which it occurs (Sniehotta & Presseau, 2012), but others argue that context-free habit measures better estimate habitual performances across contexts (Gardner & Lally, 2012). We found limited support for the latter assumption. Some participants responded to context-free SRHI measures with reference to specific performance settings, rather than summarizing across contexts. Context-specific measures may minimize error arising from between-participant differences in interpretations. They may not, however, remove such differences; some participants added contextual information when responding to highly situation-specific items (i.e., ‘drinking a second alcoholic drink after my first alcoholic drink when in a pub or bar’). Additionally, context-specific SRHI items generated unique problems arising from item complexity, with the extra subject (‘Behaviour X *in Context Y*’) causing difficulties for some. Alternatively, context-free SRHI items might be combined with BFCS cue stability indicators (Norman & Cooper, 2011). Both combinative approaches risk compounding problems associated with component indices. Another method might require participants to tailor their responses to self-identified contexts. This would, however, preclude reliable interpretation of pooled responses across participants and depends on participants accurately perceiving contextual covariates of behaviour, which our data suggest may not occur. Further, comparative work is needed to determine the most valid and sensitive measure of context-specific automatic action.

It may be beneficial to specify behaviours more carefully when assessing habit. Participants differed in their interpretations of ‘commuting by public transport’: For some, it referred to waiting at a bus stop, whereas for others, being aboard a moving bus. ‘Commuting’ denotes an elaborate sequence of sub-behaviours (e.g., leaving home, walking to the bus stop, waiting for the bus, boarding the bus, finding a seat). It seems feasible that each, but not necessarily all, of these may proceed automatically in response to contextual cues (i.e., habitually); boarding the bus may be automatic, but finding a seat may require reasoned deliberation. Detailed specification of behavioural targets is needed to enhance the conceptual clarity of applications of habit measures and to avoid conflating automatic and reflective processes. This in turn raises important questions about the application of habit within health psychology. Habit has traditionally been used to refer to the cognitive mechanism by which simple actions, such as pulling levers, proceed reflexively and in a fixed sequence (Tolman, 1932; Watson, 1913). Yet, studies have applied the SRHI to broad categories of health behaviours, such as ‘exercising’ or ‘eating healthily’ (Lucas *et al*., 2008; Verplanken & Melkevik, 2008), which are likely to be more flexibly structured (Maddux, 1997). The habit concept, as traditionally conceived, may fail to capture the complex psychological processes that regulate these behaviours. Further theoretical work might more precisely locate the role of habit in determining health behaviour.

Study limitations must be acknowledged. Firstly, only a minority of segments were problematic, suggesting that self-reporting habit may not always incur difficulties. The proportion of problem segments observed (10%) echoes results from previous ‘think-aloud’ studies; for example, French *et al*. (2007) found 9% of responses to Theory of Planned Behaviour items to be problematic. Our small sample makes it difficult to gauge the frequency with which problems may arise in larger samples. However, we sought to reveal the potential for response difficulties, rather than the probability of their occurrence. Secondly, participants typically had weak-to-moderate habits for the four behaviours, and so we may have failed to capture problems for those for whom habit is most relevant. Replications of this study might usefully focus on participants who frequently perform relevant actions in consistent settings, to ensure better representation of those with strong habits. Thirdly, we cannot be sure whether theoretical saturation was achieved. Our sample was highly educated, and so we may not have tapped problems that may arise from administering the measures to demographically diverse populations. Nonetheless, our findings testify to the potential for errors even among well-educated respondents and may capture problems underlying many recent studies of habit and health behaviour, which have been predominantly based on student samples (see Gardner *et al*., 2011). Further work, using larger samples, is, however, required to investigate the generalizability of our results to both non-student and student populations. Lastly, it is unclear whether ‘think-aloud’ narratives capture the thoughts that precede responses, or post-hoc rationalizations (cf. Nisbett & Wilson, 1977). This problem may perhaps be accentuated when applying ‘think-aloud’ methods to habit, because it is not known whether participants have sufficient insight into the thought processes that precede non-reflective actions. It is also possible that ‘thinking aloud’ may have increased attention to questions, so prompting responses that may differ from those obtained in normal conditions.

Our study suggests that multiple problems can arise in participants’ responses to the two most commonly used self-report habit measures. Although problems were only expressed in a minority of responses, it would seem prudent for researchers to take steps to mitigate such problems where possible.

## References

[b1] Adriaanse MA, Oettingen B, Gollwitzer PM, Hennes EP, de Ridder DTD, de Wit JBF (2010). When planning is not enough: Fighting unhealthy snacking habits by mental contrasting with implementation intentions (MCII). European Journal of Social Psychology.

[b2] Ajzen I (2002). Residual effects of past on later behavior: Habituation and reasoned action perspectives. Personality and Social Psychology Review.

[b3] Ajzen I (2006). http://people.umass.edu/aizen/tpb.html.

[b4] Bargh JA, Wyer RS, Srull TK (1994). The four horsemen of automaticity: Awareness, intention, efficiency, and control in social cognition. Handbook of social cognition.

[b5] Darker CD, French DP (2009). What sense do people make of a theory of planned behaviour questionnaire? A think-aloud study. Journal of Health Psychology.

[b6] Davis CG, Thake J, Vilhena N (2010). Social desirability biases in self-reported alcohol consumption and harms. Addictive Behaviors.

[b7] Eagly AH, Chaiken S (1993). The psychology of attitudes.

[b8] Eccles MP, Hrisos S, Francis JJ, Stamp E, Johnston M, Hawthorne G, Hunter M (2011). Instrument development, data collection, and characteristics of practices, staff, and measures in the Improving Quality of Care in Diabetes (iQuaD) Study. Implementation Science.

[b9] French DP, Cooke R, McLean N, Williams M, Sutton S (2007). What do people think about when they answer Theory of Planned Behaviour questionnaires? A ‘think aloud’ study. Journal of Health Psychology.

[b10] Gardner B (2009). Modelling motivation and habit in stable travel mode contexts. Transportation Research. Part F, Traffic Psychology and Behaviour.

[b11] Gardner B (2012). Habit as automaticity, not frequency. European Health Psychologist.

[b12] Gardner B, Abraham C, Lally P, de Bruijn GJ (2012). Towards parsimony in habit measurement: Testing the convergent and predictive validity of an automaticity subscale of the Self-Report Habit Index. International Journal of Behavioral Nutrition and Physical Activity.

[b13] Gardner B, de Bruijn GJ, Lally P (2011). A systematic review and meta-analysis of applications of the Self-Report Habit Index to nutrition and physical activity behaviours. Annals of Behavioral Medicine.

[b14] Gardner B, de Bruijn G-J, Lally P (2012). Habit, identity, and repetitive action: A prospective study of binge-drinking in UK students. British Journal of Health Psychology.

[b15] Gardner B, Lally P (2012). Does intrinsic motivation strengthen physical activity habit? Modeling relationships between self-determination, past behaviour, and habit strength. Journal of Behavioral Medicine.

[b16] Honkanen P, Olsen SO, Verplanken B (2005). Intention to consume seafood – the importance of habit. Appetite.

[b17] Ji MF, Wood W (2007). Purchase and consumption habits: Not necessarily what you intend. Journal of Consumer Psychology.

[b18] Kaklamanou D, Armitage CJ, Jones CR (2013). A further look into compensatory health beliefs: A think aloud study. British Journal of Health Psychology.

[b19] Kline P (1999). The handbook of psychological testing.

[b20] Lally P, van Jaarsveld CHM, Potts HWW, Wardle J (2010). How are habits formed: Modelling habit formation in the real world. European Journal of Social Psychology.

[b21] Lucas T, Alexander S, Firestone I, Lebreton JM (2008). Just world beliefs, perceived stress, and health behavior: The impact of a procedurally just world. Psychology and Health.

[b22] Maddux JE (1997). Habit, health, and happiness. Journal of Sport & Exercise Psychology.

[b23] McDaniel MA, Einstein GO (2000). Strategic and automatic processes in prospective memory retrieval: A multiprocess framework. Applied Cognitive Psychology.

[b24] McGowan L, Cooke LJ, Gardner B, Beeken R, Croker H, Wardle J Healthy feeding habits: Efficacy results from a cluster-randomized, controlled exploratory trial of a novel, habit-based intervention with parents. American Journal of Clinical Nutrition.

[b25] Nisbett RE, Wilson TD (1977). Telling more than we can know: Verbal reports on mental processes. Psychological Bulletin.

[b26] Norman P, Cooper Y (2011). The theory of planned behaviour and breast self-examination: Assessing the impact of past behaviour, context stability and habit strength. Psychology and Health.

[b27] Orbell S, Verplanken B (2010). The automatic component of habit in health behavior: Habit as cue-contingent automaticity. Health Psychology.

[b28] Ouellette JA, Wood W (1998). Habit and intention in everyday life: The multiple processes by which past behavior predicts future behavior. Psychological Bulletin.

[b29] Rhodes R, de Bruijn G-J, Matheson DH (2010). Habit in the physical activity domain: Integration with intention temporal stability and action control. Journal of Sport & Exercise Psychology.

[b30] Rothman AJ, Sheeran P, Wood W (2009). Reflective and automatic processes in the initiation and maintenance of dietary change. Annals of Behavioral Medicine.

[b31] Sniehotta FF, Presseau J (2012). The habitual use of the Self-Report Habit Index. Annals of Behavioral Medicine.

[b32] Tolman EC (1932). Purposive behavior in animals and men.

[b33] Triandis H (1977). Interpersonal behavior.

[b34] Verplanken B, Melkevik O (2008). Predicting habit: The case of physical exercise. Psychology of Sport and Exercise.

[b35] Verplanken B, Myrbakk V, Betsch T, Haberstroh S, Rudi E (2005). The measurement of habit. The routines of decision making.

[b36] Verplanken B, Orbell S (2003). Reflections on past behavior: A self-report index of habit strength. Journal of Applied Social Psychology.

[b37] Watson JB (1913). Psychology as the behaviorist views it. Psychological Review.

[b38] Wood W, Neal DT (2007). A new look at habits and the habit-goal interface. Psychological Review.

[b39] Wood W, Quinn JM, Kashy DA (2002). Habits in everyday life: Thought, emotion, and action. Journal of Personality and Social Psychology.

[b40] Wood W, Tam L, Witt MG (2005). Changing circumstances, disrupting habits. Journal of Personality and Social Psychology.

